# Fabrication, Investigation, and Application of Light-Responsive Self-Assembled Nanoparticles

**DOI:** 10.3389/fchem.2019.00620

**Published:** 2019-09-12

**Authors:** Juan Pang, Ziyu Gao, Huaping Tan, Xincheng Mao, Jialing Xu, Jingyang Kong, Xiaohong Hu

**Affiliations:** ^1^School of Material Engineering, Jinling Institute of Technology, Nanjing, China; ^2^Biomaterials for Organogenesis Laboratory, School of Materials Science & Engineering, Nanjing University of Science & Technology, Nanjing, China

**Keywords:** nanoparticle, self-assemble, light-responsive property, nanocarrier, drug delivery

## Abstract

Light-responsive materials have attracted increasing interest in recent years on account of their adjustable on-off properties upon specific light. In consideration of reversible isomerization transition for azobenzene (AZO), it was designed as a light-responsive domain for nanoparticles in this research. At the same time, the interaction between AZO domain and β-cyclodextrin (β-CD) domain was designed as a driving force to assemble nanoparticles, which was fabricated by two polymers containing AZO domain and β-CD domain, respectively. The formed nanoparticles were confirmed by Dynamic Light Scattering (DLS) results and Transmission Electron Microscope (TEM) images. An obvious two-phase structure was formed in which the outer layer of nanoparticles was composed of PCD polymer, as verified by ^1^HNMR spectroscopy. The efficient and effective light response of the nanoparticles, including quick responsive time, controllable and gradual recovered process and good fatigue resistance, was confirmed by UV-Vis spectroscopy. The size of the nanoparticle could be adjusted by polymer ratio and light irradiation, which was ascribed to its light-response property. Nanoparticles had irreversibly pH dependent characteristics. In order to explore its application as a nanocarrier, drug loading and *in vitro* release profile in different environment were investigated through control of stimuli including light or pH value. Folic acid (FA), as a kind of target fluorescent molecule with specific protein-binding property, was functionalized onto nanoparticles for precise delivery for anticancer drugs. Preliminary *in vitro* cell culture results confirmed efficient and effective curative effect for the nanocarrier on MCF-7 cells.

## Introduction

As a fundamental backbone, materials play an irreplaceable role in the development of science and technology in all fields, especially in fast-growing current functional and intelligent research fields. Stimuli-responsive materials have attracted great interest due to their adjustable properties in response to external stimuli (Guragain et al., 2015; Lorenzo et al., 2015; Cao and Wang, 2016; Lu et al., 2016; Mao et al., 2016; Ahmed et al., 2017; Huang et al., 2018; Kydd et al., 2018). From commonly used external stimuli sources including photo, heat, solution pH value, electric signal and magnetic signal, photo-responsive materials are commonly employed in bio-related fields and information-related fields on account of convenient control, high efficiency and low cost (Guragain et al., 2015; Lorenzo et al., 2015; Cao and Wang, 2016; Lu et al., 2016; Mao et al., 2016; Ahmed et al., 2017; Huang et al., 2018; Kydd et al., 2018). Photo molecular switch offered precise photo response upon specific light source enabling a material response (Beharry and Woolley, 2011; Sun et al., 2012; Pang et al., 2015; Bian et al., 2016; Ye et al., 2016; Kathan and Hecht, 2017). Currently, many molecules with precise alterable spatiotemporal structures, such as chiral helicene, azobenzene, diarylethene, spiropyran, and binaphthyl compounds, have been explored for application as photo switches (Sun et al., 2012; Yuan et al., 2014; Kim et al., 2015; Lin et al., 2016). Generally, these molecules possess conjugated structures, which influence their compatibility to aqueous environment and future application in that environment (Sun et al., 2012; Yuan et al., 2014; Kim et al., 2015; Lin et al., 2016). Moreover, photo switch cytotoxicity may restrict application in bio-related fields (Pang et al., 2018). In order to improve the performance of photo switches in aqueous environments, and decrease photo switch cytotoxicity, copolymer and polysaccharide derivatives with azobenzene pendent groups were designed, respectively, in our previous work. The former showed a more controllable light-response and light-recovery performance in an aqueous environment (Pang et al., 2018, 2019).

Although the emergence of macromolecule photo switches broaden their potential application, especially in biomedical fields, a suitable carrier is needed to realize the actual application of the functional material. Films, particles, fibers and 3D scaffolds are main forms of carriers, while nanoparticles are effective for their application in the drug delivery field (Bhosale et al., 2018; Intasa-Rad and Ogawa, 2018; Zhang et al., 2018; Ma et al., 2019). Therefore, a light responsive nanoparticle based on previous synthesized azobenzene copolymer was designed in this research.

As a drug carrier, biocompatibility and drug encapsulated capacity are two primary requirements of carrier materials (Wang et al., 2017, 2018). In consideration of the two requests, β-cyclodextrin (β-CD) possesses good biocompatibility and hydrophobic cavity, which can form an inclusion complex with small molecules (Wang et al., 2015; Zhou et al., 2015; Wang and Wu, 2016). The hydrophobic cavity of β-CD could include an azobenzene group to form a super molecular link as an effective and efficient method to prepare carriers like hydrogels and nanoparticles (Hu and Gong, 2016; Hu et al., 2017; Malik et al., 2017; Song et al., 2017). Therefore, the self-assembly technique based on interactions between β-CD group and azobenzene group was used to prepare nanoparticles in this work. Since more than one functional group per molecule is a premise of super molecular self-assemble, polymerization or crosslinking of β-CD should be obtained before fabrication of nanoparticles.

Targeted delivery is a requirement of drug delivery vehicles in order to realize effective and efficient drug delivery (Huang et al., 2017; Merzel et al., 2017). Folic acid can be recognized and integrated by a folic acid receptor, which exists in both normal tissue and tumors (Huang et al., 2017; Merzel et al., 2017). Nevertheless, either the number or activity of folic acid receptors in tumors is higher than that in normal tissue (Huang et al., 2017; Merzel et al., 2017). Thus, folic acid was used to functionalize the surface of nanoparticles during the fabrication process for the preliminary application evaluation of a nanoparticle as a drug carrier in the research. In order to realize the object, we synthesized FA functional poly(β-cyclodextrin) (PCD) through the chemical coupling method, which was assembled with copolymer and azobenzene pendent (HANN copolymer). The final functional nanoparticle was preliminarily evaluated by the cytotoxicity of cancer cells in order to explore its application in the anticancer drug delivery field.

In summary, aiming at responsive nanoparticles for the drug delivery field, we designed a light-responsive azobenzene (AZO) domain for copolymers as a stimuli-responsive unit and a self-assemble unit simultaneously. Poly(β-CD) was used to construct a nanoparticle by a driving force coming from the interaction between AZO domain and domain considering its good biocompatibility and drug controllable performance in the research. Finally, FA functionalization broadened the application of the nanoparticle in the biomedical field.

## Experiment Section

### Materials

Folic acid (FA), β-cyclodextrin (β-CD), sodium periodate, dichloromethane (DCM), diethyl ether, tetrahydrofuran (THF), dioxane, benzoyl peroxide (BPO), triethylamine (TEA), and dimethylsulfone (DMSO) were obtained from Sinopharm Chemical Reagent Co., Ltd, China. N-hydroxysuccinimide (NHS), acryloyl chloride and p-aminoazobenzene (AZO) were purchased from Aladdin. Trypsin, Dulbecco's modified Eagle's medium (DMEM), Camptothecin (CPT) and 3-(4, 5-dimethyl) thiazol-2,5-dimethyl tetrazolium bromide (MTT) were obtained from Sigma. Fetal bovine serum (FBS) was purchased from Sijiqing biotech. Co., China. All other reagents and solvents were of analytical grade and used as received.

### Synthesis of HANN Copolymer and PCDs

HANN copolymer was synthesized by copolymerization of functional monomers including HEMA, double carbon modified NHS (NAS), NVP and double carbon modified AZO, the structure of HANN was characterized in detail in our previous work (Pang et al., 2019). Briefly, AZO and NAS monomer was synthesized by acylchlorination. Then 10 mmol monomers were dissolved by 30 ml dioxane containing 0.5 mmol BPO under nitrogen atmosphere. The sealed solution was reacted at 70°C for 24 h. Final product was precipitated by diethyl ether/THF (×3) and obtained by freeze-drying (−50°C, 7-8 Pa), which was denoted with HANN copolymer.

PCD was synthesized by epichlorohydrin crosslinking according to the previous method (Chen et al., 2015). Briefly, epichlorohydrin (4 ml) was slowly added into β-CD (2 g)/30% NaOH (10 ml) solution at 40°C. Furthermore, the mixture was heated to 60°C. After the solution became viscous, HCl was added to adjust the pH value to 7. The resultant product was dialyzed for 3 days (MW: 8 kDa) and finally obtained by freeze-drying.

Poly(β-CD)-CHO (PCD-CHO) was synthesized by the oxidation method (Ye et al., 2019). Briefly, sodium periodate solution (0.6 M, 2 ml) was added dropwise into 100 ml 1.4% w/v PCD solution (pH = 2). After a 2 h reaction at room temperature in the dark, 0.3 ml ethylene glycol was then added to inactivate any unreacted periodate. The solution was purified by dialyzing (MW: 8 kDa) and then freeze-dried to get PCD-CHO.

Poly(β-CD)-CHO-FA (PCD-CHO-FA) was synthesized by the reaction between the aldehyde group and amino group. FA was dissolved in DMSO with a final concentration of 1% w/v, into which β-CD-CHO was added with a final concentration of 1% w/v. The reaction was kept at 40°C for 24 h, and PCD-CHO-FA was also obtained by dialyzing and freeze-drying. The product was characterized by proton nuclear magnetic resonance spectrum (_1_H NMR, Bruker AV-300).

### Self-Assemble of Light Response Nanoparticle

HANN was dissolved in DMSO to obtain a HANN/DMSO solution with certain concentration. Simultaneously, PCD was dissolved in water to obtain PCD solution with 10 mg/ml. Forty microliter HANN solution and 4 ml PCD solution was mixed to obtain nanoparticle, which was further dialyzed to remove DMSO. The obtained nanoparticle was characterized by dynamic light scattering (DLS, nano ZS) and transmission electron microscope (TEM, Philips, Tecnai 12). The nanoparticle was further characterized by _1_H NMR (Bruker AV-500).

Nanoparticle for *in vitro* evaluation was formed by HANN copolymer and Poly(β-CD)-CHO-FA using the same method, which was denoted as Nano-FA 33.

### Photo Response and pH Dependent Properties of Nanoparticle

Nano 33 aqueous solution was tracked by UV spectroscopy (Cary 50). A UV lamp (10 W) with light density of 5 mW/cm^2^ was used as a photo source to induce trans-to-cis transition of AZO domain. After UV irradiation, white light of 260 μW/cm^2^ was used to induce cis-to-trans recovery at room temperature. To track structural change of molecules, real-time UV spectra as a function of irradiation time and recovery time was recorded. Repeated irradiation and recovery method were applied to demonstrate fatigue resistance of the material.

Furthermore, the effective diameter of nanoparticle in suspension as a function of time in one response-recovery circle was tracked by DLS (nano ZS).

Additionally, effective diameter of nano 33 aqueous solution as a function of pH value was recorded by DLS (nano ZS). Transparency of nano 33 aqueous solution as a function of pH value was tracked by UV spectroscopy (Cary 50). TEM images of nano 33 dried from pH 3 solution was characterized by TEM (Philips, Tecnai 12).

### Simulation and Computational Details

Molecular dynamics (MD) simulations were carried out with the forcite module in MS8.0 package.

The torsion force field function form of ^*^-N=N-^*^ was $E=\frac{1}{2}\sum_{j}\left\{B_{j}\left(1-d_{j}\cos\left[n_{j}\phi \right]\right)\right\}$. However, for simulation the trans-to-cis isomerization of AZO, modified PCFF parameters of Bj = 25, dj = 1, and nj = 1 were adopted. NVT ensemble was used, and the temperature of 298 K was controlled by Nose thermostat. Moreover, velocity Verlet algorithm was applied by a time step of 1fs.

The clusters of AZO and β-CD also optimized using G09 programs at cam-b3lyp/6-31g(d) level (Trucks et al., 2009). The interaction energies (ΔE) between AZO and β-CD were calculated, and the basis set superposition error (BSSE) was corrected by counterpoise method.

### *In vitro* Evaluation for Nanocarrier

An anticancer drug (CPT) was chosen as the model drug to be encapsulated into the above-mentioned nanoparticle during the process of nanoparticle fabrication. Briefly, CPT was dissolved in HANN copolymer/DMSO solution with a different final concentration, which was further mixed with poly(β-CD) aqueous solution, as mentioned above. The final drug encapsulated nanoparticles were obtained using this method. The encapsulated CPT amount was obtained by the difference between the added amount and precipitation amount in solution. The precipitation was collected, dissolved with certain volume DMSO and quantified by the ultraviolet spectrophotometric method using UV spectroscopy (Cary 50) at 360 nm according to the standard curve. For CPT release assay, nanoparticle suspension was dialyzed in 15 ml water solution with PBS. At appropriate intervals, 3 ml released dialysis solution was withdrawn and the absorbance at 360 nm was recorded to calculate the cumulate CPT release. Simultaneously, 3 ml fresh solution was supplemented into dialysis solution.

Besides *in vitro* drug release profiles, effects of drug encapsulated nanoparticles on viability of MCF7 cells were evaluated by MTT assay. Briefly, nanoparticles DMEM solution with different nanoparticle concentration was added into the 96-well culture plate with 80 to 90% cell confluence. At different intervals, after being supplemented with 20 μL MTT, the cells were continually cultured for another 4 h. After cell was stained by MTT, they are observed by optical microscope (IX73). At the same time, 200 μL of DMSO was added to dissolve the formed formazan pigment. The absorbance of 150 μL above solution at 570 nm was recorded by a microplate reader (Tecan M200 Pro).

### Statistical Analysis

Data were analyzed using the *t*-test for differences. Results were reported as means ± standard deviation. The significant level was set at *p* < 0.05.

## Results and Discussion

### Self-Assemble of Light Response Nanoparticle

**Figure 1:**
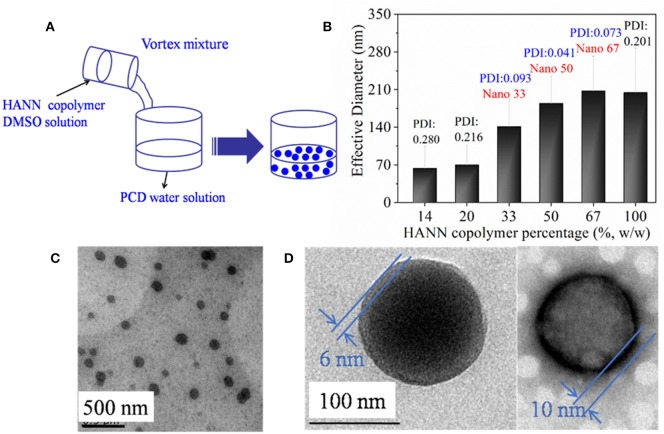
**(A)** Schematic illustration to show the assembled nanoparticle formation; **(B)** effective diameter of nanoparticle as a function of HANN copolymer percentage. TEM images of nano 33 **(C)** and **(D)** magnified images including normal image (left) and background staining image (right).

Since HANN copolymer had little solubility in water, nanoparticles were self-assembled under the help of DMSO cosolvent in water (Figure 1A). The formed nanoparticles were characterized by DLS and TEM in Figures 1B–D. In order to optimize two polymer ratio, effective diameter as a function of HANN copolymer concentration was recorded by DLS (Figure 1B). It was found that the effective nanoparticle diameter increased until the nanoparticles consisted of 67% HANN copolymer. The PDI value of effective nanoparticle diameter increased when HANN copolymer concentration was either higher than 67% or lower than 33%, which indicated the correlation between results and actual diameter became worse. Since small diameter and narrow dispersity of nanoparticles were preferred to obtain homogeneous dispersion as nanocarrier, nano 33 was chosen for further investigation. Well-dispersed nanoparticles with diameters of about 100 nm were observed by TEM analysis (Figure 1C) for nano 33, which confirmed the formation of nanoparticles by self-assembly. The diameters from the TEM image were smaller than effective diameter of 140 nm from the DLS result, which might be attributed to nanoparticle hydration in solution. In addition, distinctive light and dark color with an obvious boundary was found in a magnified unstained TEM image (Figure 1D). A dark ring with thickness of about 10 nm was witnessed in magnified background staining TEM image, which was considered as the border of nano 33. In the polysaccharide component, the samples need to be negatively stained in the process of TEM so can the morphology of PCD can be observed in white color. In this case, the dark ring was considered as the edge of nano 33 and the white surface was PCD. Combining the normal and background staining TEM images, the structure nano 33 we could refer to was a two-phase structure with PCD as the outside layer.

**Figure 2:**
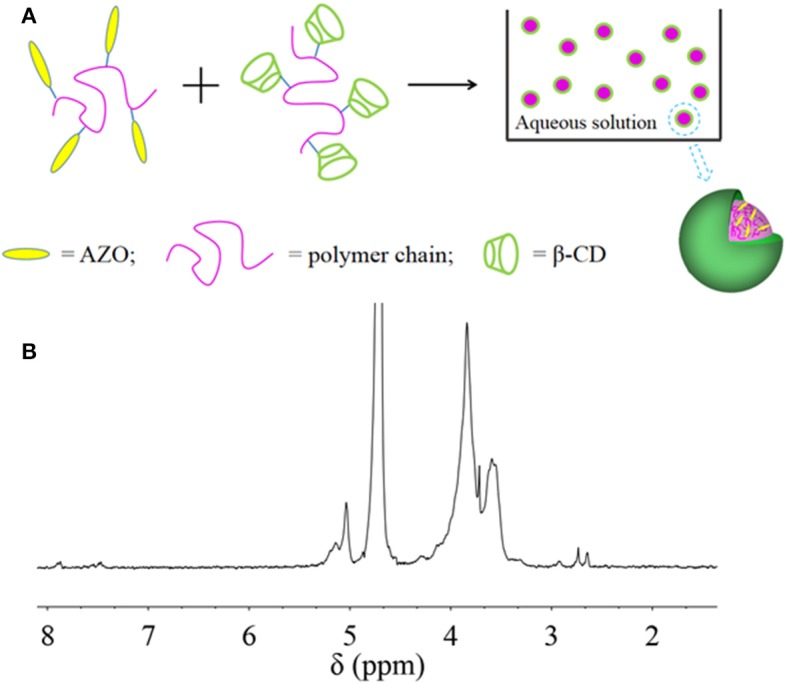
**(A)** Proposed mechanism for nanoparticle self-assembly; **(B)** ^1^H NMR spectrum of Nano 33.

From these results, it was inferred that HANN copolymer was embedded inside the nanoparticle and PCD was assembled outside the nanoparticle, which might be ascribed to the enhanced hydrophilicity or PCD rather than HANN copolymer (Figure 2A). For further structure confirmation, ^1^H NMR analysis of nanoparticles was performed (Figure 2B). The chemical shifts from 3.5 to 4.0 ppm are attributed to the protons of pyranose ring of β-CD. Simultaneously, the typical chemical shifts of 7.4 to 8.5 ppm are ascribed to the AZO domain, 2.7 ppm to the NAS domain, 1.2 to 2.3 ppm to the NVP domain and 0.5 to 1.2 ppm to the HEMA domain. These specific peaks from HANN copolymer decreased significantly, compared to the ^1^H NMR spectra of pure HANN copolymer, which was available in our previous work (Pang et al., 2019). This obvious change was attributed to the hydrophilic PCD component, which was cycled around the outer layer of nanoparticles and illustrated the inter-mechanism in the nanoparticles. The result of ^1^HNMR spectrum for nanoparticles further verified our proposed mechanism.

### Photo Response and pH Dependent Properties of Nanoparticle

**Figure 3:**
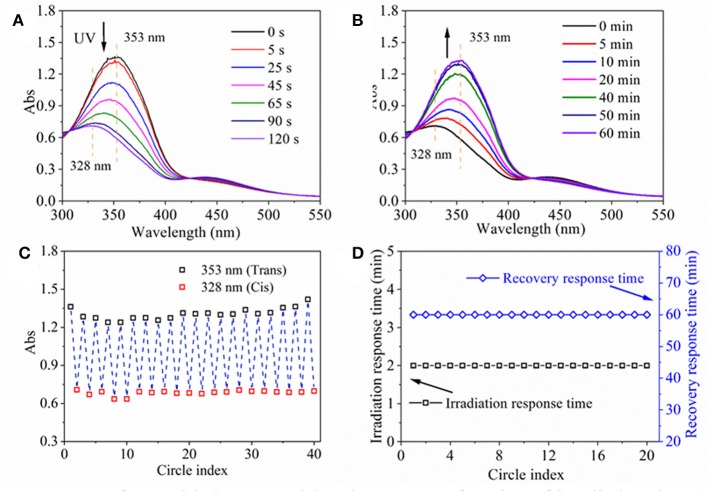
UV spectra of assembled nanoparticle aqueous solution as a function of irradiation time **(A)** and recovery time **(B)**. **(C)** Absorbance at 353 nm/328 nm of assembled nanoparticle aqueous solution, and **(D)** irradiation response time under UV irradiation and recovery response time under 260 μW/cm^2^ white light at room temperature as a function of circle index.

Firstly, UV-Vis spectra of assembled nanoparticle aqueous solution as a function of irradiation time and recovery time were obtained in order to investigate their light response process (Figures 3A,B). Before UV irradiation, a maximum absorption peak at 353 nm and a small flat absorption peak at 450 nm emerged in the UV spectrum of nanoparticle solution (Figure 3A), which was consistent with the UV spectrum of HANN copolymer from our previous research (Pang et al., 2019). Upon UV irradiation, the maximum absorbance at 353 nm decreased significantly and shifted to 328 nm and the absorbance at 450 nm increased slightly with irradiation time until 120 s on account of trans-to-cis transformation (Figure 3A). The phenomenon was similar to that of HANN copolymer except prolonged response time from 60 s to 120 s, which might be a result of confined molecular movement. Upon white light irradiation, two peaks were gradually recovered to their respective original absorbance value, in 1 h on account of cis-to-trans recovery (Figure 3B). Upon repeated UV/white light irradiation, the absorbance at 353 nm/328 nm of the nanoparticle solution as a function of cycle number was recorded to characterize fatigue resistance of nanoparticle, which was shown in Figure 3C. Simultaneously, maximum absorbance at 353 nm was stabilized at 1.2 to 1.5 regardless and minimum absorbance at 328 nm was stabilized at 0.6 to 0.8 regardless of cycle number (Figure 3C). At the same time, irradiation response time and recovery response time was stable at 2 and 60 min, respectively, regardless of cycle number (Figure 3D). These properties, including quick response time, controllable and gradual recovered process and good fatigue resistance, ensured that efficient and effective light response property are highly desirable for the nanoparticles.

**Figure 4:**
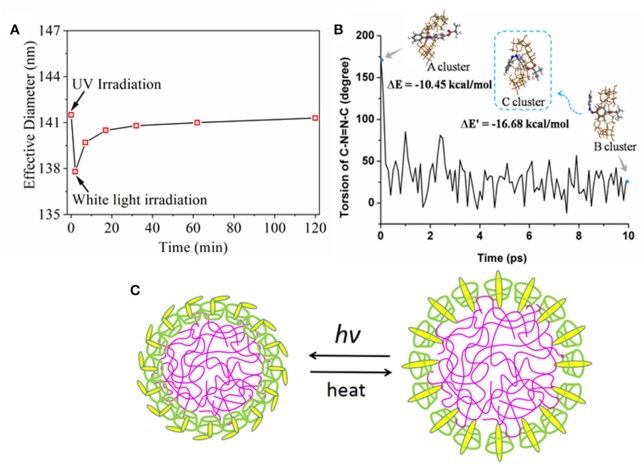
**(A)** Effective diameter as a function of irradiation time and recovery time; **(B)** Torsion of C-N=N-C (degree) of AZO evolved by dynamics time (ps) carried out by MS program. The insets of A cluster was the optimized structure of trans-AZO and β-CD by G09 package, meanwhile the intial structure of our simulation; B cluster was the final structure of the simulation; and C cluster was the optimized structure of cis-AZO and β-CD cluster from B cluster; **(C)** Proposed UV response mechanism for assembled nanoparticle.

Next, the effective nanoparticle diameter as a function of irradiation and recovery time was monitored by DLS (Figure 4A). Considering that the homogeneity and dispersion of nanoparticles may affect the self-assemble behavior, in this period the time-dependent tracking was carried on the same sample every time point to observe the influence of UV-light in dynamic diameters. The effective diameter decreased to <138 nm from 141 nm after UV irradiation and then the effective diameter was gradually increased to 141 nm after white light irradiation, as opposed to UV irradiation. From these results, it was inferred that the nanoparticles could form a tighter structure after UV irradiation and reversibly recover to their original state, which might be ascribed to an inter-molecular structure change between β-CD domain and AZO domain upon UV/white light. In order to clarify the light response mechanism for the nanoparticle, the trans→ cis process was modeled by MD simulation and modified PCFF parameters in Figure 4B. Before simulation, the initial structure of trans-AZO and β-CD cluster (A cluster) was optimized by G09 and used as a starting point for the MD simulations. In simulation, the angle of the C-N=N-C torsion decreased to a low value after time evolution of 10 ps, which indicated that trans-AZO changed to its cis form since the angle is a direct indicator for trans or cis structure of AZO domain. Simultaneously, the final structure of cis-AZO and β-CD cluster (B cluster) was obtained by simulation, which was a tighter cluster evolved by β-CD ring sliding from pendant AZO group to inner polymer domain on the foundation of A cluster. After simulation, B cluster was further optimized by G09 to form C cluster. Subsequently, the interaction energies (ΔE) of A cluster and C cluster calculated to −10.45 kcal/mol for the former and −16.68 kcal/mol for the latter by quantum chemistry, while also confirmed that the cluster of cis-AZO and β-CD was more stable than the cluster of trans-AZO and β-CD. Therefore, we proposed a UV/white light response mechanism in Figure 4. In their natural state, nanoparticles were assembled by inclusion interaction between AZO pendant group and hydrophobic cavity of β-CD domain. Upon UV light irradiation, the AZO pendant group stretched out of hydrophobic cavity so that the whole nanoparticle became more tightly bond. The mechanism was supported by both experimental and theoretical results.

**Figure 5:**
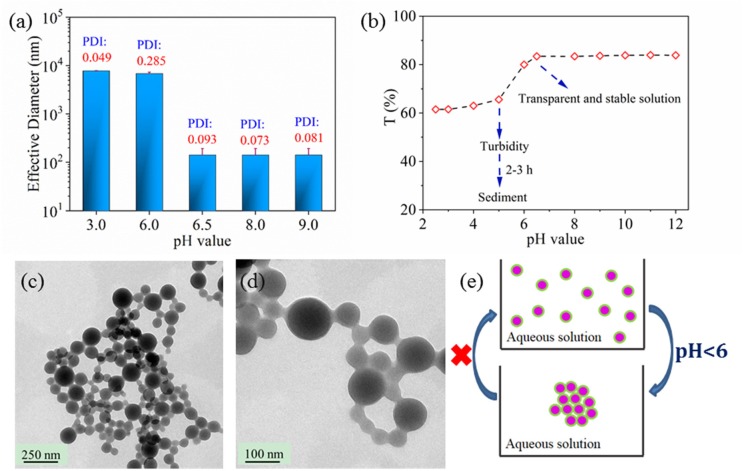
Effective diameter **(a)** and transparency **(b)** of nano 33 aqueous solution as a function of pH value. TEM images of nano 33 dried from pH 3 solution, scale bar 250 nm **(c)**, 100 nm **(d)**. Proposed pH dependent behavior for nanoparticle **(e)**.

Beside the UV response, the nanoparticles pH response was also was studied in Figure 5. Effective nanoparticle diameter increased a from 140 nm to 7 μm when the pH value of environment was < 6 according to Figure 5a. The effective nanoparticle diameter exhibited no dependent relationship on the solution pH value when the pH value was lower than 5.5 or higher than 6.5. Similar phenomenon of critical pH point for solution transparency existed in Figure 5b. At the same time, nano 33 solution was clear when pH value was higher than 6.5 or it was turbid when pH value was lower than 5.5 either from eyesight or from transparency of Figure 5b. Not surprisingly, enlarged nanoparticle size impeded light transmission so that the solution became turbid even sediment, which was assumed to be a result of nanoparticle aggregation or nanoparticle coalescence. In order to clarify the actual status of nanoparticle in acid solution, nanoparticles dried from acid solution were characterized by TEM in Figures 5c,d. On the TEM image, aggregated nanoparticles were dispersed homogeneously, which verified that pH dependent properties were due to nanoparticle aggregation. However, effective diameter kept to 7 μm and the nanoparticle solution kept turbid when the nanoparticle solution was adjusted to neutral or alkaline value again from original acid value, which indicated that nanoparticle aggregation was irreversible. Therefore, the pH dependent behavior was proposed in Figure 5E. The aggregation for nanoparticles respond in an acid environment, while their reversible process could not be realized. The pH dependent aggregation could ensure the nanoparticle staying in low pH area to play their therapeutic and responsive role, which is important for their application especially in the drug delivery field.

### *In vitro* Evaluation for Nanocarrier

**Figure 6:**
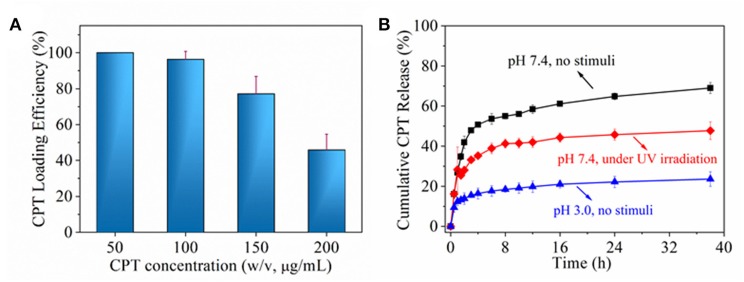
**(A)** CPT loading efficiency as a function of CPT concentration. **(B)** cumulative CPT release under different environment.

Next, the chemotherapeutic CPT was loaded into nanoparticles through the *in situ* fabrication method (Hu et al., 2016). It was found that CPT loading efficiency decreased with the increase of CPT concentration, especially when >100 μg/ml, which was shown in Figure 6A. Actually, since the loaded CPT amount was due to definite free volume of inner nanoparticle, saturated CPT loading amount made its loading efficiency decrease for its higher loading concentration. According to the result of Figure 6A, CPT loading concentration of 100 μg/ml was chosen for further drug release behavior investigation (Figure 6B). CPT molecules in nanoparticle might exist in two statuses. One is a free molecule status; the other is a combined molecule status by interaction with nanoparticle. The free molecule could be easily released from nanoparticle by diffusion mechanism; while combined molecule should be exchanged to release medium by stronger interaction from molecules, ions or even groups. In PBS without any stimuli, about 50% CPT was quickly released from nanoparticles in 1 h and another 20% CPT was gradually released within 36 h. In PBS with UV irradiation, about 30% CPT was quickly released from nanoparticle in 1 h and another 20% CPT was gradually released within 36 h. In medium of pH 3 without any stimuli, <20% CPT was gradually released from nanoparticle within 36 h. The initial burst release might be attributed to the diffusion of free CPT molecule and further gradual release might be interactions between CPT and polymer domains including β-CD domain. In PBS without any stimuli, incompact structure of nanoparticle permitted more free volume that accommodate a freer CPT molecule. Upon UV irradiation, firmer nanoparticle structure reduced free volume, which decreased free CPT molecule. While in acid medium, CPT diffusion became very slow on account of small surface area induced by aggregation of nanoparticle. To sum up, the results indicated that drug could be hold or controlled to stay a specific place through the control of stimuli light or pH value.

**Figure 7:**
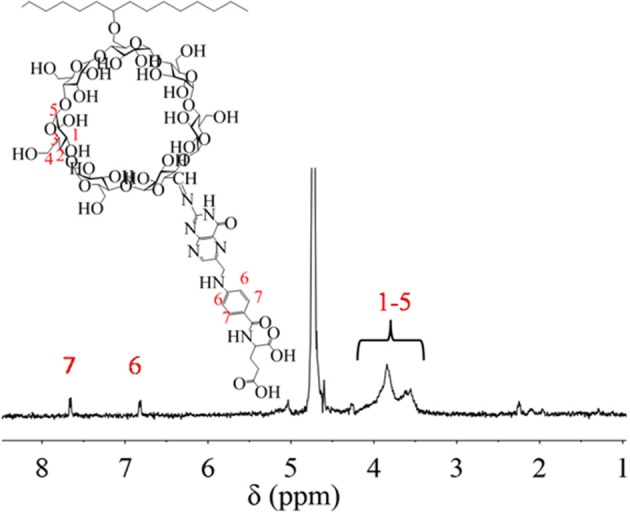
^1^H NMR spectrum of PCD-CHO-FA.

In order to increase the recognition of nanoparticle, FA was grafted onto PCD through the two-step method of oxidation and covalent crosslinking. After functionalization, PCD-CHO-FA was characterized by _1_H NMR spectrum in Figure 7. The details of chemical shift are listed as follows: chemical shifts from 3.5 to 4.2 ppm are attributed to the protons of pyranose ring on β-CD domain at 1-5 position, chemical shifts at 6.8 ppm and 7.7 ppm are attributed to the protons of benzene ring on new modified FA domain at 6 and 7 positions.

**Figure 8:**
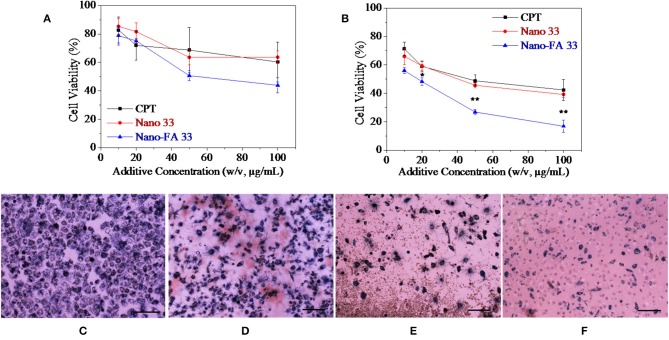
Cell viability as a function of cocultured additive concentration after **(A)** 12 h and **(B)** 24 h. Optical microscopic images of cells on TCPs control **(C)** and with 100 μg/ml **(D)** CPT, **(E)** Nano 33, and **(F)** Nano-FA 33 after cultured 24 h. Cell seeding density is 2*10^4^/well. Cells were stained by MTT. The scale is 100 μm.

Then nanoparticles fabricated by self-assemble of HANN copolymer and PCD-CHO-FA functional polymer for further *in vitro* evaluation. Two kinds of nanocarriers with or without FA targeted domain were evaluated by *in vitro* cancer cell culture along with pure CPT group and TCPs control group, and the 3 results are shown in Figure 8. After coculture for 12 h, cytoviability decreased to 70% of original value with the increase of CPT concentration, and cytoviability decreased to 70% of original value with the increase of Nano 33 concentration, while cytoviability decreased to lower than 50% of original value with the increase of Nano-FA 33 concentration (**Figur 8A**). Although no significant difference was found between the three groups, the group for Nano-FA 33 exhibited relatively good effects to inhibit cell growth. After coculture for 24 h, cytoviability decreased to lower than 50% of original value with the increase of CPT concentration, and cytoviability decreased to 45% of original value with the increase of Nano 33 concentration, while cytoviability decreased to only 10% of original value with the increase of Nano-FA 33 concentration (Figure 8B). Cell growth was nearly completely inhibited by the Nano-FA 33 nanocarrier when its concentration reached 100 μg/ml after coculture for 24 h, which showed a significant difference between the Nano-FA 33 group and other two groups. These results were further supported by MTT stained optical microscopic cell images after coculture for 24 h. On TCPs control, homogenous dispersed cells were intact with fewer cell debris (Figure 8C). On CPT group, less intact cells and more cell debris were found, which indicated more dead cells (Figure 8D). On Nano 33 group, a small number of cells were witnessed to anchor on the culture plate, which was fewer than two above-mentioned groups (Figure 8E). On Nano 33-FA group, only limited several cells were observed with a large amount of cell debris (Figure 8F). In a word, many dead cells confirmed the effectiveness and efficiency of nanocarriers on cancer cells.

## Conclusion

Nanoparticles were successfully self-assembled by HANN copolymer and PCD polymer. The effective diameter was influenced by the HANN/PCD ratio. Optimal HANN copolymer concentration was from 33 to 67%. Homogeneous dispersed nanoparticle with two-phase structure was also confirmed by TEM images. ^1^HNMR spectroscopy confirmed the outer layer of nanoparticle to be composed of PCD polymer. UV spectrum verified the efficient and effective light response property for the nanoparticle, for example quick responsive time, controllable and gradual recovered process and good fatigue resistance, which were induced by the structural change for AZO domain. Upon UV irradiation, the self-assembled nanoparticles became more compact. MD simulations found that the β-CD slides toward the HANN copolymer main chain and quantum chemistry calculated that the cluster with cis-form had even larger interaction energy than that with trans-form. MD and quantum chemistry gave a reasonable explanation of the diameter response of nanoparticles upon UV light. Nanoparticles could irreversibly aggregate in acid medium (pH < 6.0), which indicated the pH dependent characteristic for nanoparticles. The *in vitro* drug release profile confirmed that the drug could be held or controlled to stay a specific site through the control of stimuli including light or pH value. FA functionalized nanoparticles were successfully prepared for their application as a drug carrier. Preliminary *in vitro* cell culture results confirmed efficient and effective curative effect for the nanocarrier on MCF-7 cells.

## Data Availability

All datasets generated for this study are included in the manuscript/supplementary files.

## Author Contributions

JP designed macromolecule and finished the calculation part. ZG prepared and characterized the self-assembled nanoparticles. HT helped with characterization and suggested potential application for this nanoparticle. XM, JX, and JK worked for the UV light responsive behavior. XH gave the ideas and designed the whole research. All the authors were involved in the data analysis.

### Conflict of Interest Statement

The authors declare that the research was conducted in the absence of any commercial or financial relationships that could be construed as a potential conflict of interest.
